# Rare Metastatic Pathways of Renal Cell Carcinoma: Duodenal Involvement and Therapeutic Challenges

**DOI:** 10.1002/ccr3.70589

**Published:** 2025-06-30

**Authors:** Supriya Peshin, Faizan Bashir, Nagaishwarya Moka

**Affiliations:** ^1^ Department of Internal Medicine Norton Community Hospital Virginia USA; ^2^ School of Medicine Shiraz University of Medical Sciences Shiraz Iran; ^3^ Department of Hematology and Medical Oncology Appalachian Regional Health Middlesboro USA

**Keywords:** atypical metastasis of RCC, duodenal metastasis, metastatic renal cell carcinoma, mRCC, rcc

## Abstract

Duodenal metastasis from renal cell carcinoma (RCC), though rare, warrants consideration in patients with unexplained gastrointestinal (GI) symptoms. Endoscopic evaluation and immunohistochemistry (IHC) are essential for accurate diagnosis, while molecular profiling helps identify mutations influencing disease behavior and treatment response. Systemic therapy with targeted agents and immunotherapy requires careful monitoring due to potential toxicity. A multidisciplinary approach remains essential for optimizing management and surveillance strategies in metastatic RCC.

**Abbreviations**
CT: Computed tomography
EGD: Esophagogastroduodenoscopy
GI: Gastrointestinal
ICI: Immune checkpoint inhibitor
IHC: Immunohistochemistry
mRCC: Metastatic renal cell carcinoma
PBRM1: Polybromo 1
PD‐L1: Programmed death‐ligand 1
PET: Positron emission tomography
PPI: Proton pump inhibitor
RCC: Renal cell carcinoma
SBRT: Stereotactic body radiation therapy
SETD2: SET domain containing 2
TKI: Tyrosine kinase inhibitor
TMB: Tumor mutational burden
VEGF: Vascular endothelial growth factor
VHL: Von Hippel–Lindau

## 1 Introduction

Renal cell carcinoma (RCC) accounts for approximately 2%–3% of all adult malignancies and is characterized by a high metastatic potential [1]. While RCC commonly metastasizes to the lungs, bones, liver, and brain, gastrointestinal (GI) metastases, particularly to the duodenum, are exceptionally rare [2]. In fact, duodenal metastasis from RCC is so infrequent that it is reported in only a handful of cases, making it a diagnostic challenge. The duodenum's rich vascular supply and proximity to the pancreas and biliary system make it an unusual site for metastatic spread, posing significant diagnostic and therapeutic challenges.

RCC metastases can present insidiously, frequently mimicking primary GI malignancies or benign conditions such as peptic ulcer disease. As a result, a high index of suspicion is required to identify metastatic lesions in patients with a history of RCC. Immunohistochemistry (IHC) and molecular profiling play critical roles in confirming the diagnosis and guiding treatment decisions, particularly in the era of targeted therapies and immunotherapy.

Duodenal metastases from RCC are rare, requiring thorough diagnostic workup and multidisciplinary management. This report details a case that highlights the importance of including RCC in the differential diagnosis of GI lesions, even years after initial treatment. Clinicians must maintain vigilance when patients with a history of RCC present with GI symptoms, as metastases can manifest in rare locations even years after apparent remission. Moreover, personalized therapies guided by molecular profiling can offer significant benefits in managing such complex cases.

## 2 Case History

A 73‐year‐old male with a history of stage IV RCC presented with iron deficiency anemia and melena 5 years after initial diagnosis. His oncologic history began in December 2018 when imaging revealed a left renal mass with ipsilateral adrenal involvement, subsequently diagnosed as clear cell RCC (T3a, N0, M1). Histopathological examination classified the tumor as Fuhrman grade 3, indicating moderate to poor differentiation. Following radical left nephrectomy, the patient was placed on active surveillance.

In August 2020, surveillance imaging identified a 7 mm right middle lobe lung nodule, which was biopsy‐proven as metastatic RCC (positive for CD10 and CD56; negative for chromogranin, TTF1, CK7, and Pax‐2). He subsequently underwent stereotactic body radiation therapy (SBRT) for the lung metastasis with an excellent response. In October 2020, systemic therapy with pembrolizumab (200 mg IV every 21 days) was initiated, but it was discontinued in May 2021 due to grade 2 immune‐related gastritis. Axitinib (5 mg PO BID) was briefly started but discontinued due to chest discomfort.

### 2.1 Examination and Investigations

**FIGURE 1:**
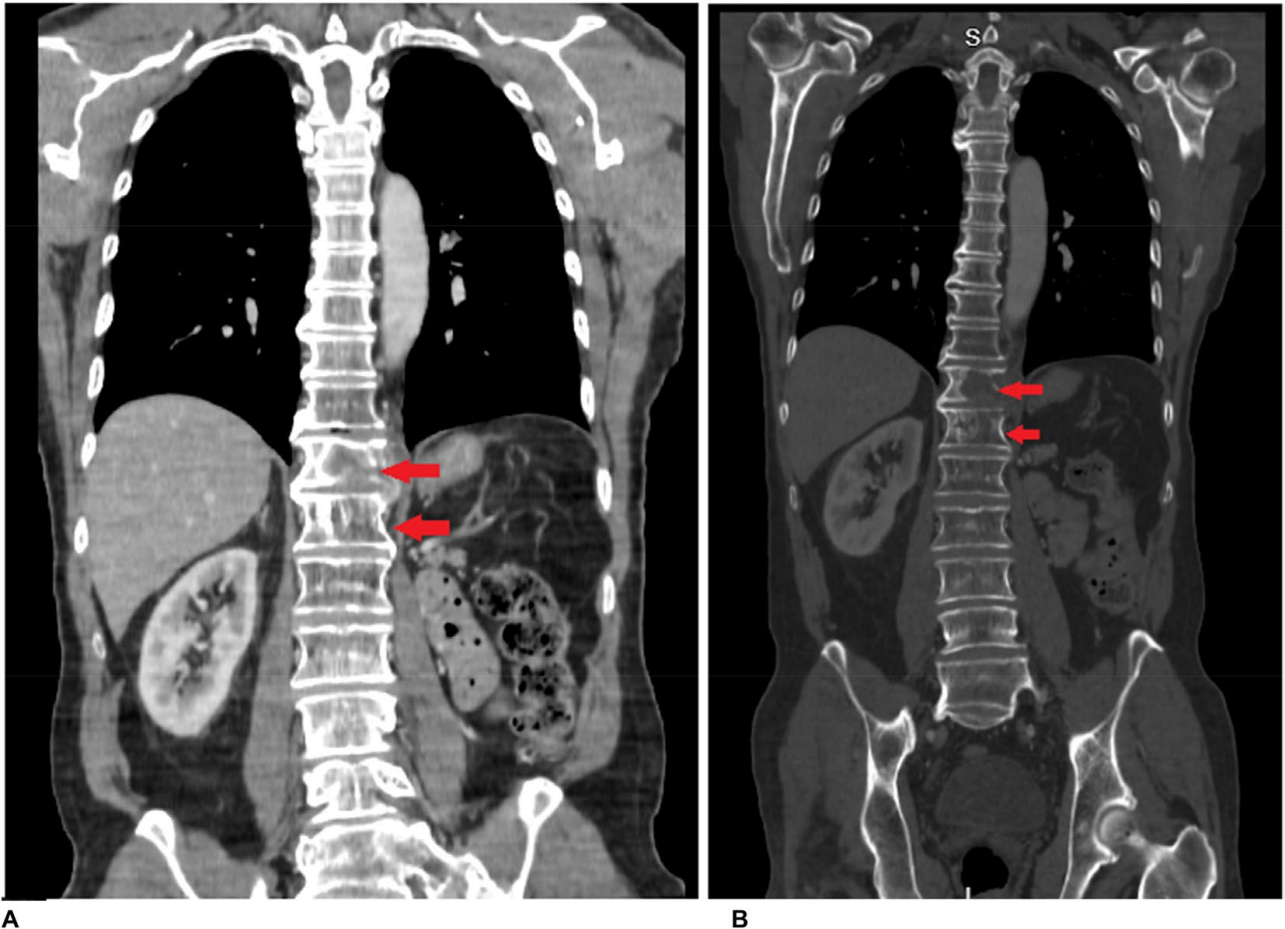
(A, B) MRI of the thoracic spine reveals hypointense vertebral lesions on T1‐weighted imaging and hyperintense or mixed intensity on T2/STIR sequences. These findings suggest bone marrow infiltration with tumor involvement, with a potential for epidural extension, increasing the risk of spinal cord compression.

**FIGURE 2:**
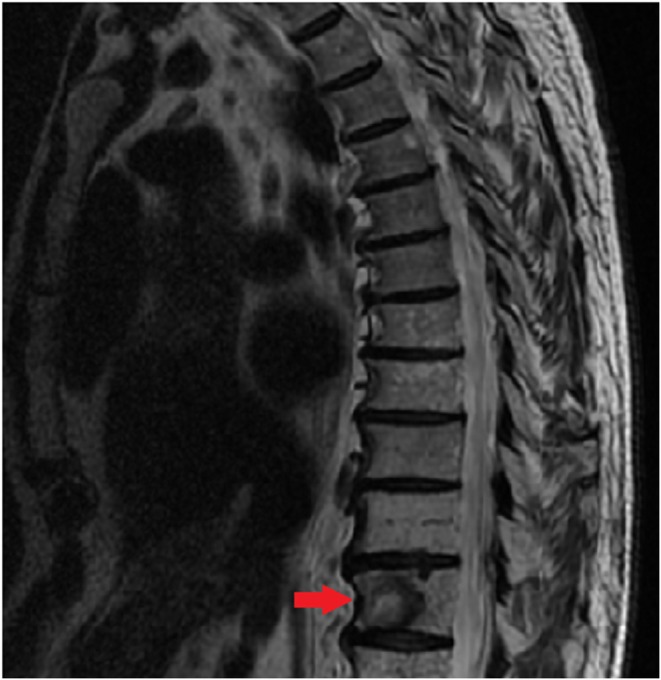
Coronal view of bone metastasis demonstrating lytic destruction of the T12 vertebral body, a characteristic feature of RCC metastases, which are typically osteolytic. There is a potential for cortical breakthrough and an increased risk of pathological fracture, with no evident sclerosis, aligning with the expected radiologic behavior of RCC.

Serial CT and PET imaging revealed no evidence of residual or metastatic disease except for stable small lung nodules and a left paraspinal mass at T12 (3.1 x 2.7 cm) (Figures 1 and 2).

In May 2023, the patient presented with melena and was found to have a duodenal ulcer on esophagogastroduodenoscopy (EGD). Histopathology confirmed metastatic clear cell RCC, with IHC demonstrating positivity for CK AE1/AE3, PAX8, CD10, EMA, and vimentin and negativity for CD3 and CD20.

Molecular profiling identified mutations in VHL, PBRM1, and SETD2, genes frequently associated with RCC metastases. The VHL mutation, a hallmark of clear cell RCC, is known to contribute to tumor invasion of vascular structures, potentially explaining this unusual metastatic pattern. Additionally, tumor mutational burden (TMB) of 4 and PD‐L1 expression of 1% suggested moderate immune evasion potential, which may influence the patient's response to immunotherapy. Germline testing for von Hippel–Lindau (VHL) syndrome was negative.

### 2.2 Management

Recurrent GI toxicity complicated the patient's treatment, necessitating the discontinuation of axitinib and temporary suspension of pembrolizumab. He was managed with oral iron supplementation for anemia and continued on cabozantinib, a multikinase inhibitor. However, cabozantinib was also held due to significant GI toxicity, particularly diarrhea, highlighting the delicate balance between efficacy and tolerability in systemic RCC therapy.

Follow‐up CT and PET scans demonstrated no evidence of residual or metastatic disease, except for the duodenal lesion, which healed following treatment. Despite the challenges with therapy‐related toxicity, the patient remained clinically stable and active at his most recent follow‐up. Notably, the resolution of his duodenal lesion underscores the potential of targeted therapy in managing rare metastatic RCC presentations.

The diagnosis of duodenal metastases in RCC presents unique challenges, necessitating close collaboration among oncologists, gastroenterologists, radiologists, and pathologists. A multidisciplinary approach is essential for optimizing treatment, surveillance, and symptom control in rare metastatic cases.

**FIGURE 3:**
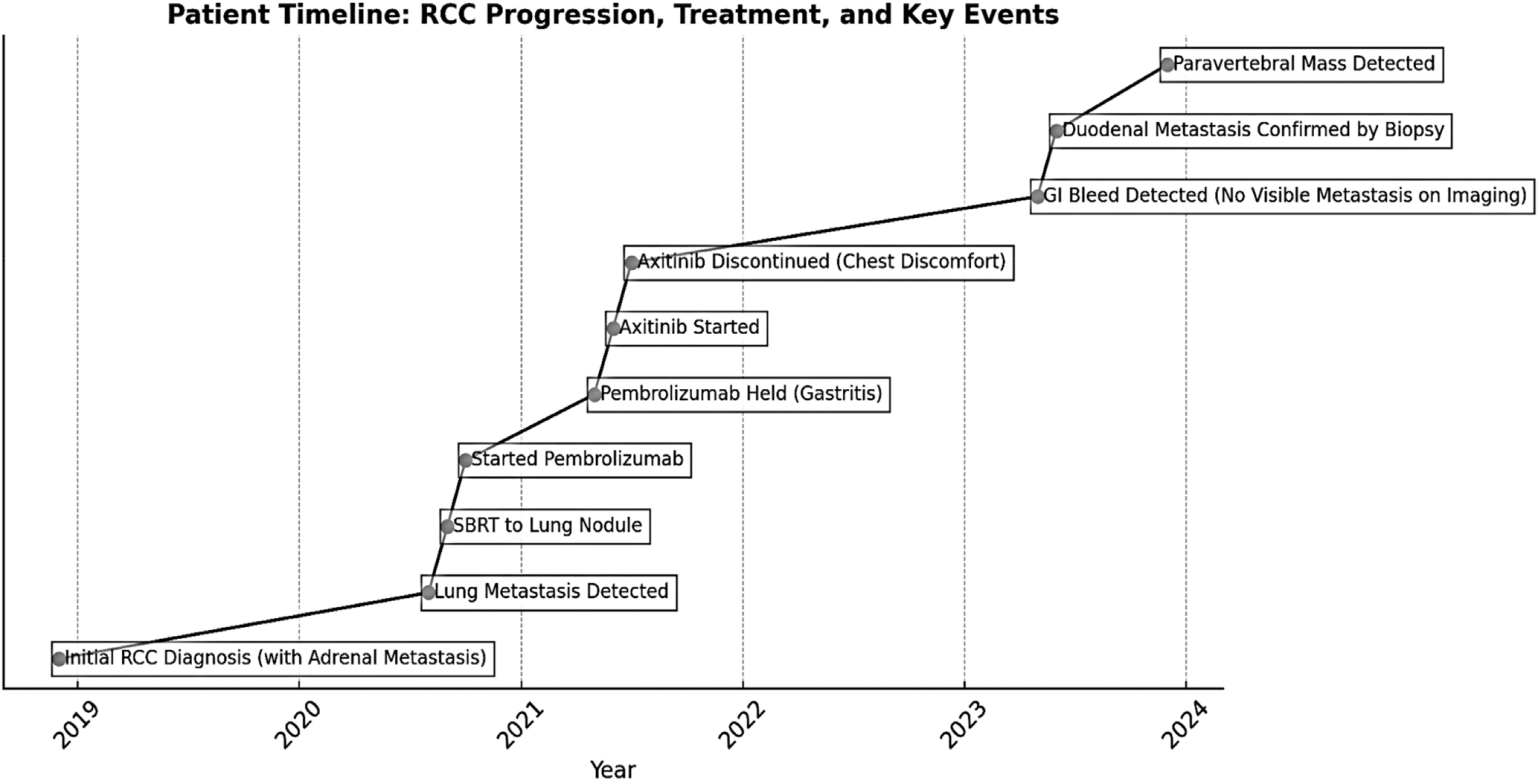
Illustrates the patient's clinical timeline, detailing the initial diagnosis, treatment milestones, disease progression, and therapeutic modifications over time.

The clinical course and treatment timeline of the patient are summarized in Figure 3.

## 3 Discussion

This case describes a 73‐year‐old male with stage IV clear cell RCC who initially presented with a left renal mass and ipsilateral adrenal metastasis. Following radical nephrectomy and SBRT for a solitary lung metastasis, he remained on active surveillance for several years. However, he later developed iron deficiency anemia and melena, prompting an EGD, which revealed a duodenal ulcerated mass.

While RCC is well known for its high metastatic potential, it most commonly spreads to the lungs, bones, liver, and brain. GI involvement is exceptionally rare, and duodenal metastasis is even less common, with only a handful of cases reported in the literature [2]. In a retrospective study by *Rony et al*., which analyzed 660 patients with metastatic RCC (mRCC), only 1.6% developed gastrointestinal metastases (GIMs), and 50% of these were in the duodenum [3]. These findings underscore the exceptional nature of our case, particularly considering the absence of visible metastases on imaging at the time of GI bleeding, further complicating diagnosis. This further emphasizes the importance of endoscopic evaluation and biopsy in RCC patients with unexplained GI symptoms, even when imaging does not initially reveal metastases.

The differential diagnosis for our patient's GI bleeding and anemia initially included more common conditions such as peptic ulcer disease, NSAID‐induced gastritis, or primary GI malignancies. However, endoscopic biopsy of the duodenal ulcer confirmed metastatic clear cell RCC, which was unexpected given the absence of detectable metastases on imaging.

IHC was critical in confirming the renal origin of the tumor. The biopsy specimen was positive for CK AE1/AE3, PAX8, CD10, EMA, and vimentin, which are hallmark markers of clear cell RCC [4]. PAX8 positivity confirmed the renal origin of the tumor, as this marker is not typically expressed in primary GI malignancies. Coexpression of CD10 and vimentin, both associated with RCC, further supported the diagnosis, reinforcing the need to consider metastatic RCC in patients with GI lesions [5].

In a multicenter retrospective study of 74 patients with RCC and GIMs conducted by *Carneiro et al*., GIMs were found to often present with anemia or GI bleeding, typically occurring a median of 1.9–5.4 years after the initial RCC diagnosis. The study highlighted that diagnosis frequently requires endoscopic evaluation and that management varies based on location and symptoms [6], particularly in cases where imaging alone can be inconclusive. Similarly, a single‐center study of 660 metastatic RCC cases, including 11 with digestive metastases, by Rony emphasized that unexplained anemia or persistent GI symptoms in RCC patients should prompt endoscopic evaluation, especially in the era of molecular‐targeted therapies and immunotherapy [3].

Given the unusual metastatic site, molecular profiling was performed to assess the tumor's genetic characteristics. The patient's tumor demonstrated mutations in VHL, PBRM1, and SETD2, which are commonly associated with clear cell RCC [7] and have significant implications for tumor behavior and therapy selection. The VHL gene is frequently altered in RCC and plays a role in angiogenesis regulation via the hypoxia‐inducible factor (HIF) pathway [7]. The loss of VHL function leads to increased VEGF expression, which may explain this patient's response to VEGF‐targeting therapies like axitinib and cabozantinib. Additionally, the PBRM1 mutation, associated with immune modulation, has been linked to responses to immune checkpoint inhibitors (ICIs). The presence of this mutation in our patient may have influenced his initial response to pembrolizumab before it was discontinued due to toxicity [8]. Furthermore, SETD2 loss has been linked to genomic instability and poor prognosis [9], which could potentially explain the unusual metastatic pattern in this patient, including the development of duodenal and later paravertebral metastases.

These molecular findings guided our therapeutic approach, providing a strong rationale for VEGF‐TKI therapy and ICI, the current standard of care for metastatic RCC [10].

The patient initially received pembrolizumab (PD‐1 inhibitor) in combination with axitinib (VEGFR‐TKI), a regimen established to provide synergistic efficacy in metastatic RCC [11]. However, treatment was complicated by toxicity. Pembrolizumab was held due to grade 2 immune‐related gastritis, requiring proton pump inhibitor (PPI) therapy and close monitoring [12]. Axitinib was subsequently discontinued due to chest discomfort, necessitating a switch in systemic therapy [13]. To manage disease progression, cabozantinib (a multikinase VEGFR‐TKI) was introduced, given its effectiveness in second‐line therapy for metastatic RCC. However, treatment was once again complicated by persistent diarrhea, leading to temporary treatment holds [14]. This case illustrates the therapeutic challenges in balancing efficacy with toxicity, especially in patients with prior GI complications. The management of RCC metastases must be highly individualized, weighing treatment tolerability against disease control.

The prognosis of RCC patients with GIMs remains highly variable, with a median survival of 19 months in the GETUG study and 12 months in the single‐center study [3], [6]. Notably, patients with glandular metastases (adrenal, pancreas, thyroid) had longer survival outcomes, which is relevant to our patient who initially presented with an adrenal metastasis [3].

The timeline of duodenal metastasis varies significantly across cases. In our patient, duodenal involvement was detected nearly 5 years after RCC diagnosis, whereas in the literature, considerable variation exists. Geramizadeh [2] reported metastasis 16 years postnephrectomy, one of the longest reported delays. Rustagi [15] described metastatic RCC to the duodenum occurring 13 years after nephrectomy, with a presentation of severe upper GI bleeding and anemia. Tanaka [16] reported concomitant pancreatic and duodenal metastases 12 years postnephrectomy, demonstrating that RCC metastases can occur in multiple distant sites even after prolonged remission. In contrast, Bhatia [17] described a 55‐year‐old male who presented with jaundice and an abdominal lump just 1‐year postnephrectomy, where the duodenal lesion was initially suspected to be a primary GI tumor, but endoscopic biopsy confirmed metastatic RCC.

Managing metastatic RCC to the duodenum presents significant challenges. Recent clinical trials have demonstrated that combination therapy with ICIs and VEGF‐targeted agents improves outcomes, though treatment‐related toxicities remain a concern. The KEYNOTE‐426 trial established pembrolizumab + axitinib as an effective first‐line regimen, while the CheckMate‐9ER trial confirmed the efficacy of nivolumab + cabozantinib. However, as observed in our patient, treatment‐related toxicities remain a major challenge, often necessitating therapy modifications or discontinuation. Studies have reported that up to 75.8% of patients on combination regimens experience grade 3–4 toxicities, requiring careful monitoring and individualized treatment adjustments [11].

## 4 Conclusion

This case highlights the critical importance of implementing a personalized, multidisciplinary approach to balance efficacy with tolerability, particularly for patients exhibiting uncommon metastatic patterns. As treatment strategies evolve, additional research is essential to optimize combination regimens while minimizing adverse effects, thereby improving outcomes for patients with complex metastatic RCC presentations. Given the late onset and potential for recurrence, long‐term surveillance is critical, even in patients with stable disease. In our case, the paravertebral mass detected after the duodenal metastasis further supports the need for ongoing monitoring.

## Author Contributions

**Supriya Peshin:** conceptualization, data curation, investigation, methodology, project administration, supervision, writing – original draft. **Faizan Bashir:** data curation, formal analysis, visualization, writing – review and editing. **Nagaishwarya Moka:** project administration, supervision.

## Consent

Written informed consent was obtained from the patient to publish this report in accordance with the journal's patient consent policy.

## Conflicts of Interest

The authors declare no conflicts of interest.

## Data Availability

The authors have nothing to report.
